# Atypical mitral valve infective endocarditis presenting with fever and left upper abdominal pain due to splenic infarction: a case report

**DOI:** 10.3389/fmed.2026.1878305

**Published:** 2026-08-14

**Authors:** Binlong Zhang, Xuefeng Lin, Haocheng Zhao

**Affiliations:** 1 Department of Gastroenterology, Yueqing People’s Hospital, Yueqing, Zhejiang, China; 2 Department of Clinical Laboratory, Yueqing People’s Hospital, Yueqing, Zhejiang, China; 3 Department of Hematology, Yueqing People’s Hospital, Yueqing, Zhejiang, China

**Keywords:** abdominal pain, infective endocarditis, splenic infarction, streptococcal bacteremia, transesophageal echocardiography

## Abstract

Infective endocarditis (IE) may present with non-specific systemic symptoms and embolic complications, making early diagnosis challenging, particularly when initial transthoracic echocardiography (TTE) is non-diagnostic. We report a case of atypical mitral valve IE in a 40-year-old man who presented with persistent fever and left upper abdominal pain rather than overt cardiac symptoms. On admission, inflammatory markers were elevated, whereas emergency chest and abdominal computed tomography showed no definite acute infectious focus. Initial TTE demonstrated only mild mitral and tricuspid regurgitation, with no definite vegetation. However, bedside cardiac auscultation identified a grade 3–4/6 systolic murmur at the mitral area, prompting further investigation. Contrast-enhanced abdominal magnetic resonance imaging subsequently revealed splenic infarction, indicating an embolic event. Blood cultures from both aerobic and anaerobic bottles grew *Streptococcus sanguinis*. In the setting of persistent fever, positive blood cultures, a prominent systolic murmur, and splenic embolic involvement, transesophageal echocardiography (TEE) was performed and demonstrated a 7 × 5 mm echogenic lesion on the atrial side of the mitral valve, together with moderate mitral regurgitation, supporting the diagnosis of IE. Following targeted intravenous antimicrobial therapy, the patient became afebrile, abdominal pain resolved, and inflammatory markers progressively normalized. Follow-up TEE showed persistent moderate mitral regurgitation without the previously observed abnormal echogenic lesion, and the patient was discharged in improved condition with oral antibiotics and arranged cardiology follow-up. Infective endocarditis should remain in the differential diagnosis of patients with fever of unclear origin accompanied by abdominal pain or splenic infarction, particularly when initial transthoracic echocardiography is non-diagnostic. In such settings, careful physical examination, timely blood culture acquisition, and early transesophageal echocardiography may help avoid diagnostic delay.

## Introduction

Infective endocarditis (IE) is a potentially life-threatening infection of the endocardial surface of the heart, most commonly involving the cardiac valves (1–3). In recent years, the incidence and epidemiological profile of IE have changed, partly because of aging populations, increasing healthcare-associated exposures, greater use of intracardiac devices and prosthetic valves, and other emerging risk factors affecting both older and younger patients. These changes have made the clinical presentation and diagnostic evaluation of IE increasingly complex (4). Blood culture-negative infective endocarditis also remains an important diagnostic challenge, particularly in patients who have received prior antimicrobial therapy or who are infected with fastidious or difficult-to-culture organisms. Therefore, the diagnosis of IE should not rely solely on blood culture positivity, but should integrate clinical features, embolic manifestations, microbiological data, and echocardiographic findings. Despite advances in diagnostic imaging, microbiological methods, and antimicrobial therapy, IE continues to be associated with substantial morbidity and mortality because of its heterogeneous clinical presentation and propensity for systemic complications (1, 5, 6). Diagnosis may be relatively straightforward in patients with persistent bacteremia, overt valvular dysfunction, and echocardiographically visible vegetations; in routine practice, however, the initial presentation is often non-specific and may mimic other infectious, abdominal, or inflammatory conditions (1–3). Embolic events are among the most clinically significant manifestations of IE and may involve the brain, spleen, kidneys, or peripheral vasculature, sometimes preceding definitive cardiac findings (6–8). In this setting, splenic infarction may represent an important but easily overlooked diagnostic clue, particularly in patients who present primarily with fever and abdominal pain rather than classical cardiac symptoms (8–10). Echocardiography remains central to diagnosis, but transthoracic echocardiography may be non-diagnostic in early disease or when vegetations are small, rendering transesophageal echocardiography essential in selected patients with persistent clinical suspicion (2, 11, 12).

We report a case of mitral valve IE in a 40-year-old man who presented atypically with fever and left upper abdominal pain secondary to splenic infarction.

Initial transthoracic echocardiography did not demonstrate definite evidence of endocarditis, whereas bedside cardiac auscultation identified a prominent systolic murmur, and subsequent blood cultures grew *Streptococcus sanguinis*, prompting further assessment with transesophageal echocardiography, which disclosed a mitral valvular lesion consistent with IE (13–15). This case highlights several clinically relevant issues. First, IE may initially present through embolic complications rather than overt cardiac manifestations (7, 8, 16). Second, careful physical examination and timely blood culture acquisition remain diagnostically important in patients with fever of unclear origin (13, 14, 17). Third, early transesophageal echocardiography should be pursued when clinical suspicion remains high despite a non-diagnostic transthoracic study (2, 12, 18). Taken together, this case underscores a practical point: IE should remain in the differential diagnosis of unexplained fever accompanied by left upper abdominal pain or splenic infarction, even when initial cardiac imaging is inconclusive (2, 6, 8).

## Case presentation

### Patient information

A 40-year-old man presented with a 2-day history of fever and left upper abdominal pain. The fever developed without an identifiable precipitating factor, was accompanied by chills, and reached a maximum temperature of 39.0 °C. He described paroxysmal dull pain in the left upper abdomen that worsened with activity. He denied nausea, vomiting, anorexia, chest pain, chest tightness, diarrhea, urinary symptoms, cough, or sputum production. No definite respiratory, urinary, gastrointestinal, skin, or soft-tissue source of infection was identified during the initial evaluation, and emergency chest and abdominal computed tomography did not reveal a clear infectious focus. Initial evaluation in the fever clinic showed leukocytosis with neutrophilia and an elevated C-reactive protein level (white blood cell count, 10.18 × 10^9/L; neutrophils, 82.0%; C-reactive protein, 78.72 mg/L). Emergency non-contrast computed tomography of the chest and abdomen showed no definite acute thoracic or abdominal abnormality, although low-density lesions in the liver and spleen were noted, and contrast-enhanced magnetic resonance imaging was recommended. Empirical intravenous levofloxacin was initiated, and the patient was subsequently admitted for further evaluation.

### Clinical findings and initial assessment

After admission, the patient remained febrile, with temperatures fluctuating around 38.0 °C, and continued to report intermittent left upper abdominal discomfort. Electrocardiography showed sinus rhythm with high left ventricular voltage. Initial transthoracic echocardiography demonstrated mild mitral and tricuspid regurgitation, with a preserved left ventricular ejection fraction of 70%, but no definite valvular vegetation. On physical examination, however, a grade 3–4/6 systolic murmur was clearly audible at the mitral area. No peripheral stigmata of infective endocarditis, such as petechiae, Janeway lesions, Osler nodes, splinter hemorrhages, or conjunctival hemorrhage, were documented in the available medical records. No focal neurological deficits were recorded. Intravenous cefoperazone-sulbactam was started on April 17, 2026. Repeat laboratory testing showed progressive inflammation, with a white blood cell count of 14.01 × 10^9/L, a neutrophil count of 11.09 × 10^9/L, a C-reactive protein level of 140.35 mg/L, and a procalcitonin level of 0.145 ng/mL.

### Diagnostic assessment

**Figure 1:**
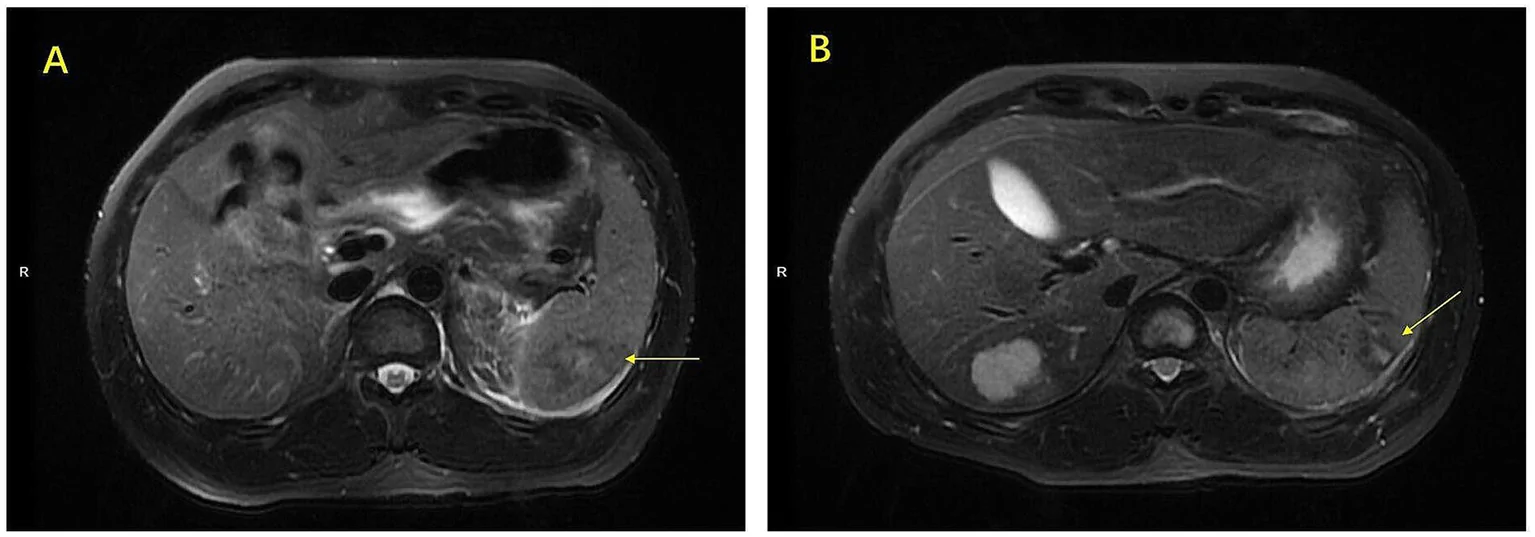
Contrast-enhanced abdominal magnetic resonance imaging of splenic infarction. **(A)** Axial upper abdominal MR image demonstrates splenomegaly with a peripheral wedge-shaped/patchy abnormal signal in the splenic parenchyma (arrow). **(B)** Corresponding contrast-enhanced axial image demonstrates a sharply demarcated non-enhancing hypoenhancing splenic lesion (arrow), with a small amount of adjacent perisplenic fluid, consistent with splenic infarction.

**Figure 2:**
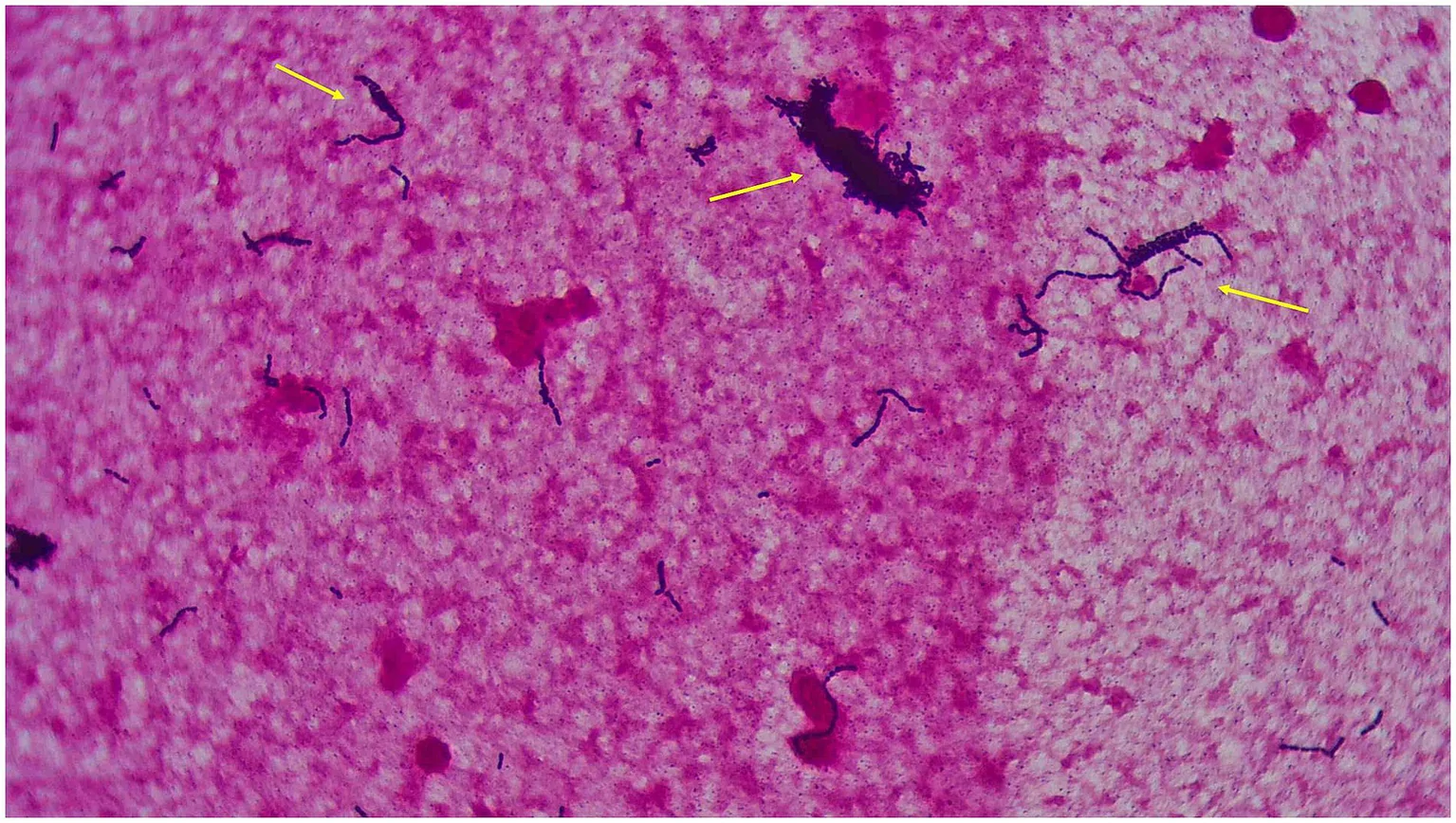
Direct Gram-stained smear from the positive blood culture bottle (original magnification ×1,000). Purple-staining coccoid organisms arranged predominantly in chains are present in the background of blood elements and stain precipitate; representative chain-forming Gram-positive cocci are indicated by arrows.

Contrast-enhanced upper abdominal magnetic resonance imaging performed on April 18, 2026 demonstrated splenomegaly with splenic infarction and a small amount of left upper abdominal fluid (Figure 1). Additional findings included hepatic hemangiomas, focal pancreatic fatty infiltration or lipoma, a left renal cyst, and an indeterminate enhancing lesion in the lower pole of the left kidney requiring dedicated evaluation. On April 19, 2026, both anaerobic and aerobic blood culture bottles collected on April 17, 2026 became positive after 17.6 and 22.6 h, respectively. The organism was identified in the original microbiological report as *Streptococcus sanguinis*, and direct Gram staining demonstrated chain-forming Gram-positive cocci (Figure 2). Antimicrobial susceptibility testing showed susceptibility to penicillin (MIC 0.064 μg/mL), ceftriaxone (MIC ≤0.25 μg/mL), and vancomycin (MIC 0.5 μg/mL). The original isolate was not preserved; therefore, additional confirmatory testing by MALDI-TOF mass spectrometry or 16S rRNA sequencing could not be performed. In the setting of persistent fever, positive blood cultures, a systolic murmur at the mitral area, and splenic infarction as an embolic clue, infective endocarditis was strongly suspected. Antimicrobial therapy was changed from cefoperazone-sulbactam to intravenous piperacillin-tazobactam, and transesophageal echocardiography was arranged.

**Figure 3:**
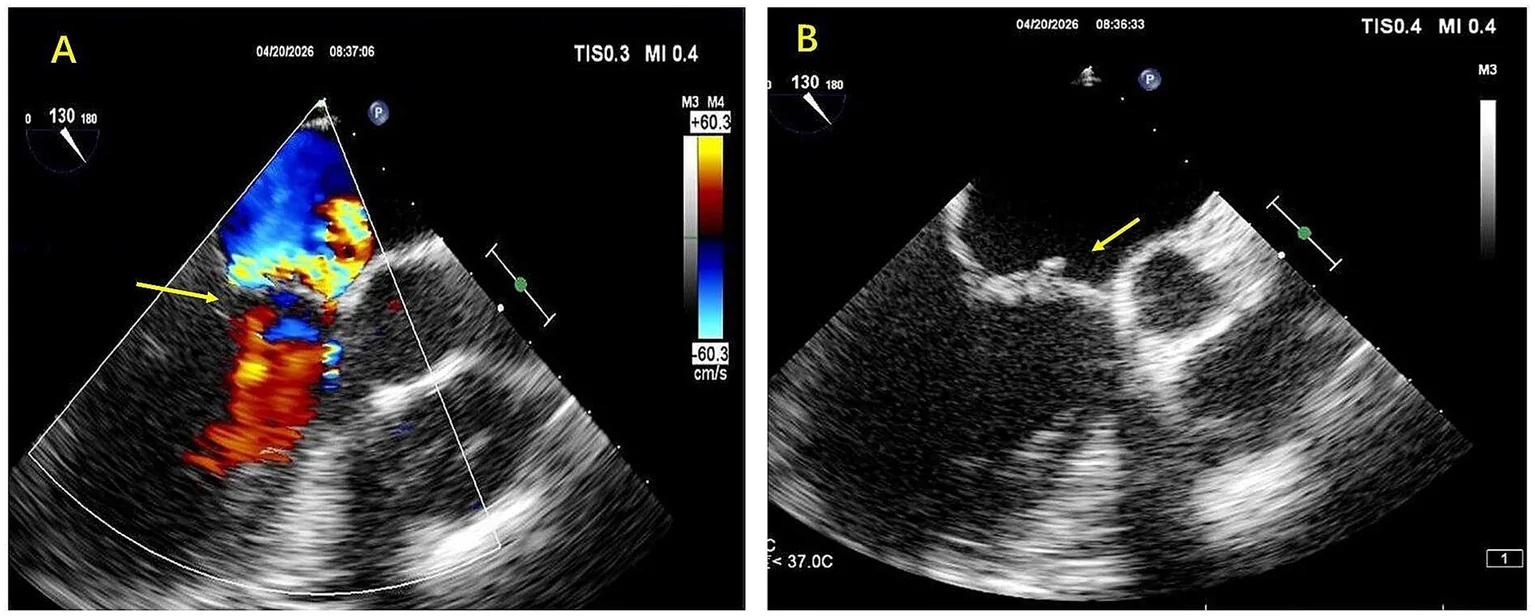
Transesophageal echocardiographic findings of the mitral valve. **(A)** Color Doppler transesophageal echocardiography demonstrates a mitral regurgitant jet directed into the left atrium (arrow). **(B)** Two-dimensional transesophageal echocardiography demonstrates a small echogenic nodular lesion attached to the atrial side of the mitral valve (arrow), suspicious for valvular vegetation.

Transesophageal echocardiography performed on April 20, 2026 demonstrated an approximately 7 × 5 mm echogenic lesion on the atrial side of the mitral valve, interpreted as possible infective endocarditis, together with moderate mitral regurgitation (Figure 3). No left atrial appendage thrombus or interatrial septal discontinuity was identified. According to the updated 2023 Duke-International Society for Cardiovascular Infectious Diseases criteria, the diagnostic criteria met in this case were mapped as follows: (1) major imaging criterion: transesophageal echocardiography demonstrated an approximately 7 × 5 mm echogenic lesion attached to the atrial side of the mitral valve, consistent with a valvular vegetation; (2) minor criterion, fever: the patient had persistent fever, with a maximum temperature of 39.0 °C; (3) minor criterion, vascular phenomenon: contrast-enhanced abdominal magnetic resonance imaging demonstrated splenic infarction, consistent with systemic embolization; and (4) minor criterion, microbiological evidence not meeting a major criterion: *Streptococcus sanguinis* was isolated from both the aerobic and anaerobic bottles of the available blood culture set. Because the available records did not document two or more independently collected positive blood culture sets, the microbiological evidence was conservatively classified as a minor microbiological criterion rather than a major microbiological criterion. Overall, the patient fulfilled one major criterion and three minor criteria, supporting a clinical diagnosis of definite infective endocarditis under the 2023 Duke-ISCVID framework.

### Therapeutic intervention

Following targeted intravenous antimicrobial therapy, the patient became afebrile by April 21, 2026, and his abdominal pain gradually improved. Follow-up laboratory testing on April 24 showed a white blood cell count of 12.00 × 10^9/L and a C-reactive protein level of 22.94 mg/L, with a normalized procalcitonin level of 0.041 ng/mL.

### Follow-up and outcomes

Repeat transesophageal echocardiography on April 29 demonstrated persistent moderate mitral regurgitation without the previously observed abnormal mitral echogenic lesion. By April 30, inflammatory markers had declined further (white blood cell count, 6.93 × 10^9/L; C-reactive protein, 7.02 mg/L). The patient was discharged on May 1, 2026, in improved condition, with an individualized post-discharge oral antimicrobial continuation plan consisting of cefuroxime axetil 250 mg twice daily and close outpatient cardiology follow-up. This post-discharge regimen reflected the real-world management of this individual patient and should not be interpreted as a guideline-recommended standard treatment regimen for streptococcal infective endocarditis. Short-term in-hospital follow-up showed clinical improvement, resolution of fever and abdominal pain, declining inflammatory markers, and disappearance of the previously observed mitral echogenic lesion on repeat transesophageal echocardiography. Outpatient cardiology follow-up was arranged after discharge; however, longer-term post-discharge follow-up data were not available in the records, which represents a limitation of this report.

### Timeline

The chronological timeline of symptoms, investigations, antimicrobial treatment, echocardiographic findings, laboratory changes, and clinical outcomes is summarized separately in Table 1. This separate timeline was included to clarify the sequence of fever onset, abdominal imaging, blood culture positivity, transthoracic and transesophageal echocardiography, antimicrobial changes, and clinical improvement.

**Table 1:** Chronological summary of the clinical course and key diagnostic findings.

Date	Key clinical events and findings	Management/implication
2026-04-14 to 2026-04-15	Fever, chills, and left upper abdominal pain; initial laboratory tests showed leukocytosis, neutrophilia, and elevated C-reactive protein; chest and abdominal computed tomography showed no definite acute abnormality	Empirical intravenous levofloxacin initiated
2026-04-16	Persistent fever after admission; electrocardiography showed sinus rhythm with high left ventricular voltage; initial transthoracic echocardiography showed mild mitral and tricuspid regurgitation without definite vegetation; grade 3–4/6 systolic murmur audible at the mitral area	Continued evaluation; blood cultures obtained
2026-04-17	Progressive inflammatory response (white blood cell count 14.01 × 10^9/L, C-reactive protein 140.35 mg/L, procalcitonin 0.145 ng/mL)	Intravenous cefoperazone-sulbactam started
2026-04-18	Contrast-enhanced abdominal magnetic resonance imaging demonstrated splenic infarction with splenomegaly and a small amount of left upper abdominal fluid	Embolic complication recognized
2026-04-19	Aerobic and anaerobic blood cultures grew *Streptococcus sanguinis*	Infective endocarditis strongly suspected; antimicrobial therapy changed to intravenous piperacillin-tazobactam; transesophageal echocardiography arranged
2026-04-20	Transesophageal echocardiography demonstrated an approximately 7 × 5 mm echogenic lesion on the atrial side of the mitral valve, with moderate mitral regurgitation	Diagnosis of mitral valve infective endocarditis supported
2026-04-21 to 2026-04-24	Became afebrile and abdominal pain improved; inflammatory markers decreased	Continued targeted intravenous antimicrobial therapy
2026-04-29 to 2026-04-30	Repeat transesophageal echocardiography showed persistent moderate mitral regurgitation without the previously observed lesion; inflammatory markers declined further	Favorable short-term response
2026-05-01	Symptoms markedly improved	Discharged with oral cefuroxime axetil and outpatient cardiology follow-up

CRP, C-reactive protein.

## Discussion

This case illustrates a diagnostically important presentation of left-sided infective endocarditis (IE), in which systemic embolization rather than overt cardiac symptoms dominated the initial clinical picture (1, 3, 6). IE is well recognized to present with a broad range of manifestations, and embolic events remain among its most consequential complications (1, 6, 7). The brain, spleen, kidneys, and peripheral vasculature are commonly affected, and embolization may occasionally precede definitive cardiac imaging findings (7, 8, 16). Splenic embolism and splenic infarction are established complications of left-sided IE, yet they may be underrecognized because abdominal pain is non-specific and may initially direct attention toward primary intra-abdominal pathology rather than occult endocardial infection (8–10). In the present case, left upper abdominal pain and MRI-confirmed splenic infarction were the key extracardiac findings that redirected the diagnostic workup toward an embolic source and ultimately to mitral valve IE. This pattern reinforces the importance of considering IE in patients with fever of unclear origin and splenic ischemic lesions, even in the absence of classical cardiac complaints (2, 8, 19). Although splenic infarction as an embolic manifestation of IE has been well described, the present case is clinically instructive because the initial presentation was dominated by fever and abdominal pain, while the first transthoracic echocardiographic study did not show definite vegetation. The educational value of this case therefore lies not in the novelty of splenic infarction itself, but in the risk of missed or delayed diagnosis when an established embolic manifestation occurs in an atypical clinical context. Compared with previously reported cases and reviews of infective endocarditis complicated by splenic embolism or splenic infarction, the present case shares the recognized pattern of left-sided endocarditis presenting with systemic embolic involvement. However, several features make this case diagnostically instructive: abdominal pain and fever dominated the initial presentation, emergency computed tomography did not identify a clear acute abdominal source, the initial transthoracic echocardiogram was non-diagnostic, and the diagnosis depended on integrating splenic infarction, Streptococcus bacteremia, a prominent mitral systolic murmur, and subsequent transesophageal echocardiographic findings. This comparison highlights that the clinical challenge is not the rarity of splenic infarction in IE, but the risk that an established embolic manifestation may be misattributed to primary abdominal disease when cardiac findings are initially inconclusive.

Another clinically relevant issue is the limited negative predictive value of a single non-diagnostic transthoracic echocardiographic study in suspected IE. Current guidelines recommend transthoracic echocardiography (TTE) as the first-line imaging modality, but transesophageal echocardiography (TEE) is recommended when TTE is negative or inconclusive despite persistent clinical suspicion; repeat TTE or TEE is also advised when the initial findings are discordant with the overall clinical picture (2, 12, 19, 20). This approach is particularly relevant in native-valve IE, in which TEE has superior sensitivity for detecting small vegetations and lesions located on the atrial aspect of the mitral valve (11, 12, 18). In this patient, the initial TTE demonstrated only mild valvular regurgitation, whereas subsequent TEE identified a small mitral lesion together with moderate mitral regurgitation, thereby establishing endocardial involvement not apparent on the initial study. The present case therefore supports a clinically important principle: a negative or non-diagnostic TTE should not provide false reassurance when microbiological, auscultatory, or embolic findings continue to suggest IE (2, 12, 18). Importantly, the absence of definitive findings on initial TTE, and in some cases even the absence of initial blood culture positivity, should not exclude IE when the overall clinical probability remains high. In such situations, repeated clinical reassessment, timely blood culture acquisition or repetition when appropriate, and escalation to TEE are essential for avoiding missed or delayed diagnosis. Non-bacterial thrombotic endocarditis (NBTE) should also be considered in the differential diagnosis of endocardial lesions or systemic embolic events, particularly when blood cultures are negative and clinical evidence of infection is limited (21). NBTE is distinct from blood culture-negative infective endocarditis and is usually associated with non-infectious prothrombotic conditions, including malignancy-associated hypercoagulability and autoimmune disorders. Libman-Sacks endocarditis represents an important form of NBTE associated with systemic lupus erythematosus and antiphospholipid syndrome. In the present case, however, persistent fever, positive blood cultures for *Streptococcus sanguinis*, a mitral systolic murmur, splenic infarction, TEE findings, and clinical improvement after antimicrobial therapy supported infective endocarditis rather than NBTE (21).

Microbiological evidence and bedside examination remain important in the contemporary diagnostic pathway. Blood cultures remain central to the diagnosis of IE and to the selection of antimicrobial therapy, and streptococcal bacteremia in a febrile patient with an embolic event should substantially increase suspicion for native-valve IE (13–15, 17). Although the modern diagnostic framework relies heavily on imaging, physical examination retains clear clinical value (13, 19). In this patient, the prominent systolic murmur at the mitral area represented a discordant but highly informative bedside finding when initial TTE was unrevealing and appropriately prompted escalation to TEE. The diagnosis therefore emerged not from any single investigation in isolation, but from integration of persistent systemic inflammation, positive blood cultures, embolic organ injury, and focused cardiac examination (2, 13, 14).

Two practical implications arise from this presentation. First, unexplained fever accompanied by left upper quadrant abdominal pain or splenic infarction should prompt active consideration of occult left-sided IE rather than being attributed solely to abdominal disease (8–10). Second, diagnostic escalation should be guided by the overall weight of clinical evidence rather than by an isolated initial imaging result (2, 12, 19). The likely source of *Streptococcus sanguinis* bacteremia was not definitively identified in this case. No clear respiratory, urinary, gastrointestinal, skin, or soft-tissue infectious focus was found during the initial evaluation. Because *Streptococcus sanguinis* is a recognized component of the normal oral flora and is a well-established cause of bacteremia after dental manipulation or minor oral mucosal trauma, an oral or dental portal of entry was considered the leading hypothesis. However, a dedicated dental or oral examination was not documented, and the portal of entry could not be confirmed retrospectively. We acknowledge that antimicrobial management is an important limitation of this case. Current guideline-based management of streptococcal infective endocarditis generally requires an organism- and susceptibility-directed antimicrobial regimen, usually with a prolonged intravenous phase. The POET trial demonstrated that, in carefully selected clinically stable patients with left-sided infective endocarditis who had received initial intravenous antimicrobial therapy, a protocolized transition to oral antimicrobial therapy was non-inferior to continued intravenous therapy. However, the application of oral step-down therapy requires strict patient selection, confirmed microbiological susceptibility, controlled infection, appropriate oral regimen selection, and structured outpatient follow-up. In the present case, the initial antimicrobial therapy was started empirically before infective endocarditis was recognized, and the post-discharge oral cefuroxime axetil continuation plan reflected individualized real-world management rather than a validated POET-style protocol. Therefore, this regimen should not be regarded as a standard, guideline-recommended, or generalizable treatment strategy for infective endocarditis (22). Therefore, the principal value of this case lies in its diagnostic message: persistent fever, splenic infarction, positive blood cultures, and a new or prominent murmur should prompt early reconsideration of infective endocarditis and escalation from non-diagnostic transthoracic echocardiography to transesophageal echocardiography. In routine practice, timely recognition often depends less on the availability of advanced tests than on avoiding premature diagnostic closure (3, 6, 19). The present case demonstrates that maintaining suspicion despite a non-diagnostic initial TTE, obtaining blood cultures early, and proceeding to second-line echocardiography when the clinical picture remains discordant may materially improve diagnostic accuracy and reduce the risk of missed systemic complications (2, 12, 13).

## Conclusion

Infective endocarditis should remain a diagnostic consideration in patients with unexplained fever accompanied by left upper abdominal pain or splenic infarction, even when initial transthoracic echocardiography is non-diagnostic. In this setting, a prominent murmur, positive blood cultures, and evidence of systemic embolization should prompt early transesophageal echocardiography and reassessment of diagnostic probability. Timely recognition may improve diagnostic accuracy and reduce the risk of missed left-sided endocardial infection and its systemic complications.

## Data Availability

The original contributions presented in the study are included in the article/supplementary material, further inquiries can be directed to the corresponding author.
